# Rectal Diagnosis in Women

**Published:** 1870-09

**Authors:** H. R. Strong


### Rectal Diagnosis in Women.

Rectal diagnosis subdivides itself at once into a three-fold group: namely,

1. The diagnosis of disease strictly rectal;

2. The diagnosis of rectal disease as originating, aggravating, or otherwise modifying uterine disorder; and

3. The diagnosis of rectal disease as the result of outlying pelvic or pelvi-abdominal lesions.

For every one of these groups, and in every instance, a direct digital examination is absolutely indicated. It should never, under any circumstances, be dispensed with.

1. The diagnosis of disease strictly rectal. For the examination of the rectum, as is well known, a variety of instruments have been employed. These have been, in the main, of two classes,

*a.* Dilators, two, or three, or many-branched, and dating back to a great antiquity; bronze instruments of the kind indicated having been found in Roman excavations; and,

*b.* True specula, of metal, or of silvered glass; these also being sometimes several-bladed.

The latter of the above classes is that most frequently employed at present. Of the several forms of instrument, Fergusson's is in the possession of almost every surgeon, and by every one, so far as

I know, it is considered indispensible in practice.

I trust I shall not be considered an idol-breaker, or an enemy to our useful coadjutors, the instrument-makers, when I say that, for purposes of diagnosis, I have long since thrown the anal speculum aside, and this no matter what its material or form. For examination of the rectum in men, the speculum is required; for examination of the rectum in women, this is not the case, and under some circumstances, just as obtains with the vaginal speculum in reference to uterine disorder, it is worse than useless, as tending to divert attention from more important points than those striking the eye.

Let me explain what I have said respecting the non-necessity of the anal speculum in women as compared with men.

By passing the finger into the vagina, and pressing it backward and downward over the levator ani, the rectum can be everted through its sphincter, like the finger of a glove. This can ordinarily be done to a very great degree; it can always be done to a certain extent. Should the sphincter be unusually irritable, and spasmodically contracting with violence when touched from below, or thus from above, it can be forcibly distended by the thumbs, and temporily ruptured, as I am in the habit of doing in such cases; the procedure above indicated thus becoming easy. We can in this manner ascertain the presence of chancre or chancroid, the character of polypi, the extent and number of internal hæmorrhoids, the position of the inner orifice in fistula, etc., with far greater certainty and alacrity than by the speculum, or can be done in the male, while the mere eversion process, provided rupture of the sphincter is not necessary, is attended by very little pain.

So far as I am aware, this suggestion of rectal eversion for diagnosis, though so simple and so effective, is an original one. It may, however, be very familiar to some of my readers, and may have been mentioned by writers. In this matter of method, every old procedure is new to many minds, or has been forgotten, and every new suggestion seems but the rediscovery of something already well known. All that I can say about it is, that I have practiced this measure for many years, that I have shown it to a great many physicians who have been present at my examinations and operations, and that none of them ever saw or heard of it before. Eversion of the recto-vaginal septum through the vulval orifice, by pressure from within the rectum, is partially possible, and a modification of it is familiar to those accoucheurs who are accustomed to hasten a fœtal head delayed at the outlet, by a finger within the mother's anus; and to some gynecologists, who have had to remove a tight fitting foreign body from within the vagina, or a three-winged intra-uterine pessary from within a nullipara. In the latter instances, one great advantage of this procedure has been by the finger in the rectum, to fix the body to be removed, and prevent its rotation during attempts at its direct grasp. The same effects have been sought, with the addition of preventing their escape upward, toward or into the sigmoid flexure, in the case of foreign bodies within the anus, by digital pressure upon the rectum from within the vagina. Mr. Clay, of Manchester, lays much stress upon this procedure, and withal urges caution, lest by thus pressing downward, the sphincter ani should be ruptured. I shall hereafter show that this is precisely what should be sought rather than avoided in such cases. These it will be perceived are different procedures from the rectal eversion from above, for diagnosis, that I have described, which in practice will be found alike effectual and gratifying, and equally so, in many cases, during operative interference. Regarding rupture of the sphincter ani, where indicated by forcible distention, I shall speak hereafter, merely premising that, to Van Buren, after Récamier, we are indebted for certain of the indications of this very valuable, and at times indispensable, proceeding. —H. R. STRONG, M. D., *in Am. Journ. Obstetrics.*

